# SHORT AND LONG-TERM RESULTS OF LAPAROSCOPIC ESOPHAGOCARDIOMYOTOMY WITH FUNDOPLICATION (HELLER-PINOTTI SURGERY) IN THE TREATMENT OF NON-ADVANCED ACHALASIA (MEGAESOPHAGUS)

**DOI:** 10.1590/0102-6720202400010e1803

**Published:** 2024-06-17

**Authors:** João Bosco CHADU, Jefferson Alvim de OLIVEIRA, Adilson Gomes FAION, Bruno ZILBERSTEIN

**Affiliations:** 1 Universidade Federal de Uberlândia, Digestive Surgery Unit – Uberlândia (MG), Brazil; 2 Faculdade São Leopoldo Mandic, Minimally Invasive Surgery, Master of Medical Sciences – Campinas (SP), Brazil.

**Keywords:** Esophageal Achalasia, Heller Myotomy, Laparoscopy, Acalasia Esofágica, Miotomia de Heller, Laparoscopia

## Abstract

**BACKGROUND:**

Videolaparoscopic esophagocardiomyotomy with fundoplication has been a widely used technique for the treatment of achalasia. This study analyzes the safety and effectiveness of the technique in the treatment of non-advanced achalasia (megaesophagus) in a Brazilian federal university public hospital.

**AIMS:**

To evaluate the short- and long-term results of videolaparoscopic treatment of non-advanced megaesophagus in a public university hospital in Brazil, employing the esophagocardiomyotomy technique with fundoplication.

**METHODS:**

The medical records of 44 patients who underwent surgical treatment for non-advanced achalasia at the Clinical Hospital of Federal University of Uberlândia (UFU-MG), Minas Gerais, from January 2001 to July 2021 were analyzed. The following data were evaluated: gender, age, etiology, radiological classification of Rezende-Alves and Ferreira-Santos, immediate and late complications (mean follow-up of 31.4 months), need or not for conversion to open access, postoperative reflux, performance or not of endoscopic esophageal dilation in the preoperative period, postoperative mortality, frequency of pre and postoperative symptoms (persistent dysphagia, regurgitation, heartburn, vomiting, odynophagia, and weight loss), surgery time, hospital stay, duration of dysphagia, pre and postoperative weight, and Eckardt score.

**RESULTS:**

Among the analyzed patients, 23 (52.3%) were male, and 21 (47.7%) were female, with a mean age of 50.8 years. No early complications were recorded and there were 27.2% cases of late gastroesophageal reflux. Postoperative weight gain was 81.8% and the success rate of surgery according to the Eckardt score was 84.1%.

**CONCLUSIONS:**

Esophagocardiomyotomy with fundoplication is an effective and safe technique for the treatment of non-advanced achalasia.

## RESUMO

**RACIONAL:**

A esofagocardiomiotomia com fundoplicatura videolaparoscópica é uma técnica amplamente utilizada para o tratamento da acalasia. Este estudo avalia se esta técnica é segura e efetiva para o tratamento da acalasia não avançada (megaesôfago) em hospital público federal universitário.

**OBJETIVOS:**

Avaliar em um hospital universitário público no Brasil os resultados imediatos e tardios do tratamento videolaparoscópico do megaesôfago não avançado pela técnica de esofagocardiomiotomia com fundoplicatura.

**MÉTODOS:**

Foram analisados retrospectivamente os prontuários de 44 pacientes submetidos ao tratamento da acalasia não avançada no Hospital de Clínicas da Universidade Federal de Uberlândia (UFU-MG) no período de janeiro de 2001 até julho de 2021. Avaliou-se: sexo, idade, etiologia, classificação radiológica de Rezende-Alves e Ferreira-Santos, complicações imediatas e tardias (seguimento médio de 31,4 meses), necessidade ou não de conversão para via aberta, refluxo pós-operatório, realização ou não de dilatação endoscópica do esôfago no pré-operatório, mortalidade pós-operatória, frequência dos sintomas no pré e pós-operatório (disfagia persistente, regurgitação, pirose, vômitos, odinofagia e emagrecimento), tempos de cirurgia, internação hospitalar, disfagia, peso pré e pós-operatório e escore de Eckardt.

**RESULTADOS:**

Entre os pacientes analisados, 23 (52,3%) eram do sexo masculino e 21 (47,7%) eram do sexo feminino, com média de idade de 50,8 anos. Não foram registradas complicações precoces e houve 27,2% de casos de refluxo gastroesofágico. O ganho de peso no pós-operatório foi de 81,8% e a taxa de sucesso da cirurgia segundo o escore de Eckardt foi de 84,1%.

**CONCLUSÕES:**

A esofagocardiomiotomia com válvula antirrefluxo por videolaparoscopia é uma técnica eficaz e segura para o tratamento da acalasia não avançada.

## INTRODUCTION

Achalasia or megaesophagus is a disease whose most common etiologies are of chagasic or idiopathic origin. It can affect any gender and age and lead to a considerable deterioration in the patient’s quality of life^2,13,26,38^.

The main symptoms are dysphagia, weight loss, regurgitation, and retrosternal pain^7,35,38^. The diagnosis is based on the clinical condition, serology in cases of Chagas disease, esophageal contrast radiography, and conventional or high-resolution esophageal manometry. Upper digestive endoscopy should be performed to rule out association with esophageal squamous cell carcinoma^19,27,29,45^.

Achalasia can be treated endoscopically by injecting botulinum toxin, pneumatic dilation, and perioral endoscopic myotomy (POEM)^33,37,41,44^. Several surgical techniques can be used for treatment, the most prevalent being laparoscopic esophagocardiomyotomy with fundoplication and, in some cases esophagectomy^8,37,43,44,45^.

The objective of this study was to evaluate, in a federal university public hospital in Brazil, the immediate and late results of the videolaparoscopic treatment of non-advanced megaesophagus, using the esophagocardiomyotomy with fundoplication technique — the Heller-Pinotti surgery.

## METHODS

This is an observational, retrospective, longitudinal, and descriptive study in which the records of patients submitted to laparoscopic surgical treatment of non-advanced megaesophagus employing the technique of esophagocardiomyotomy with fundoplication (Heller-Pinotti surgery) were analyzed. They were treated at the University Hospital of the Federal University of Uberlândia (UFU-MG) from January 2001 to July 2021. The study was approved by the Ethics Committee of Faculdade São Leopoldo Mandic under 5.129.656 and by the Ethics Committee of UFU-MG under 5.244.184.

Patients with advanced megaesophagus and non-advanced megaesophagus operated by other surgical techniques or by open access were excluded. During this period, 147 patients were operated on for achalasia: 44 videolaparoscopic esophagocardiomyotomies with fundoplication (Heller-Pinotti surgery) for non-advanced achalasia, eight videolaparoscopic esophagocardiomyotomies with fundoplication for advanced achalasia, 17 open esophagocardiomyotomies with fundoplication for non-advanced achalasia, 46 Thal-Hatafuku surgeries, 15 Thal-Hatafuku surgeries associated with longitudinal esophagectomy, 14 subtotal esophagectomies, and three Serra-Doria surgeries.

This study considered for inclusion those 44 patients who underwent esophagocardiomyotomy with videolaparoscopic fundoplication for the treatment of non-advanced chagasic and idiopathic megaesophagus.

The Heller-Pinotti surgery technique employed in the study begins with the patient in horizontal dorsal decubitus under general anesthesia. Initially, an umbilical puncture is performed with a Veress needle to create a pneumoperitoneum of 14 mmHg. Five trocars are positioned: two 10 mm trocars in the umbilicus and left hypochondrium, and three 5 mm trocars in the epigastrium, right hypochondrium, and left flank. After the passage of the trocars, the dissection and isolation of the esophagus and diaphragmatic pillars begin. Next, an extramucosal esophagocardiomyotomy of 6 cm in the esophagus and 3 cm in the stomach is performed, associated with fundoplication with three suture lines: the first in the posterior esophageal wall; the second sutured to the left border of the myotomy; and the third, covering the naked mucosa, sutured to the edges of the right side of the myotomy, as recommended by Pinotti^31,32^. In Brazil, this surgery is known as Heller-Pinotti surgery.

Data were collected through a research form prepared by the authors with an active search for information in the medical records and subsequent data analysis using the RStudio statistics software. The following categorical variables were studied: gender, etiology of achalasia, radiological classification of megaesophagus by Rezende-Alves and Ferreira-Santos^7,14,34^, early and late intraoperative and postoperative complications, need or not to convert to open access, presence or absence of postoperative reflux, indication or not for endoscopic dilation of the esophagus in the preoperative period, postoperative mortality, and frequency of the following pre and postoperative symptoms: persistent dysphagia, regurgitation, heartburn, vomiting, odynophagia, and weight loss. These variables were presented as absolute and/or relative frequency. Numerical variables studied were age, duration of preoperative dysphagia, duration of surgery, length of hospital stay, length of postoperative follow-up, and pre and postoperative weight. These variables were presented with results calculated by mean and standard deviation.

We used an association of the radiological classifications by Rezende-Alves and Ferreira-Santos to classify the degree of megaesophagus^7,14,34^. These findings are described below.

The Rezende-Alves classification is:

Group I: esophagus of normal caliber, slow transit, small contrast retention;

Group II: esophagus with mild to moderate increase in caliber, considerable retention of contrast, presence of tertiary waves, associated or not with hypertonicity of the distal esophagus;

Group III: esophagus with a great increase in diameter, reduced motor activity, hypotonia of the distal esophagus, great contrast retention; and

Group IV: dolicomegaesophagus, esophagus with great retention capacity, atonic, elongated and tortuous^34^.

The Ferreira-Santos classification is:

Grade I: moderate dilation, up to 4 cm in diameter, small stasis at 5 minutes;

Grade II: dilation up to 7 cm in diameter, stasis at 30 minutes;

Grade III: dilation up to 10 cm in diameter, esophageal sigmoid elongation, significant stasis with the presence of food residues; and

Grade IV: dilation greater than 10 cm in diameter, presence of food residues only^14^.

To evaluate the results of the treatment of achalasia, the presence of intraoperative early (30 days after surgery) and late (one year after surgery) complications was analyzed. To assess clinical improvement, the Eckardt score, weight, and presence of pre and postoperative symptoms were compared. The Eckardt score was used to evaluate the effectiveness of the treatment of achalasia based on the absence or presence and frequency of the following symptoms: weight loss, dysphagia, regurgitation, and retrosternal pain (Table 1)^11^. When the score is ≤3, it means remission of the disease (Table 1)^11^. The Eckardt score was obtained from the information found in the medical records, either with the value of the score extracted on the day of the outpatient consultation or with the same calculated based on the information found with the mean and median of the score in the pre and postoperative. It was not possible to obtain the Eckardt score in eight patients due to incomplete data in the medical records. The preoperative Eckardt score was compared with the postoperative score to assess the outcome of the surgery, using the paired Wilcoxon test, with a significance level of p<0.05. A scatter plot was performed to demonstrate the correlation between the difference in pre and postoperative weight and the postoperative Eckardt score calculated using Pearson’s correlation. The frequencies of the following pre and postoperative symptoms were also compared: persistent dysphagia, regurgitation, heartburn, vomiting, odynophagia, and weight loss. The level of statistical significance of the relationship between these symptoms was assessed using the chi-square test (p<0.05), and the magnitude of these differences, when present, was assessed using Cramer’s V test.

**Table 1:** Eckardt score^11^.

Score	Symptoms			
	Weight loss	Dysphagia	Retrosternal pain	Regurgitation
0	None	None	None	None
1	<5 Kg	Occasional	Occasional	Occasional
2	5–10 Kg	Daily	Daily	Daily
3	>10 Kg	Each meal	Each meal	Each meal

Clinical staging of achalasia – Eckardt Score: Stage 0: 0–1 – Clinical impact: remission. Stage I: 2–3 – Clinical impact: remission. Stage Il: 4–6 – Clinical impact: therapeutic failure. Stage Ill >6 – Clinical impact: therapeutic failure.

## RESULTS

The cohort included 23 (52.3%) males and 21 (47.7%) females. The mean age of the patients was 50.8 years, with a minimum age of 14 years and a maximum of 83 years. In the group of patients studied, 25 (57%) had chagasic etiology and 19 (43%) had idiopathic etiology. Patients were classified based on contrast-enhanced radiography of the esophagus using the Rezende-Alves and Ferreira-Santos’ classifications, with 22 patients (50%) grade III, 15 (34%) grade II, six (14%) grade I, and one (2%) without description in the medical record.

The mean preoperative dysphagia time was 51 months (ranging 5–240 months). The mean duration of surgery was 231 minutes (105–460 min.), mean hospital stay of 3.5 days (2–8 days), and mean postoperative follow-up time of 31.4 months (1–180 months) (Table 2).

**Table 2:** Surgery times, dysphagia, hospitalization, and follow-up.

	Average	Minimum	Maximum	Standard deviation
Surgery time	231 minutes	105 minutes	460 minutes	6,529.521
Dysphagia time	51 months	5 months	240 months	5,258.763
Length of hospital stay	3.5 days	2 days	8 days	1,453.108
Follow-up	31.4 months	1 month	180 months	2,915.741

The study showed that 30 patients (68%) were submitted to preoperative pneumatic esophageal endoscopic dilations, with nine cases having one dilation, ten cases having two dilations, seven patients having three dilations, and four with more than three dilations.

There were ten (22.7%) intraoperative complications with eight (18%) esophageal perforations, all of which were small perforations diagnosed and immediately sutured during surgery. Two cases of conversions to laparotomy were identified, one (2.2%) due to hypercapnia, and the other (2.2%) due to hepatomegaly. These complications had no clinical repercussions. No early complications (30 days postoperatively) were recorded.

Regarding late complications, patients with up to one year of follow-up were excluded, resulting in 33 patients in the late analysis. Of them, nine (27.2%) had gastroesophageal reflux in the postoperative period diagnosed by clinical symptoms, and in one case, by upper digestive endoscopy showing erosive esophagitis.

Among the 33 patients analyzed, five (15.1%) had persistent dysphagia. One of the patients with persistent dysphagia underwent endoscopic dilation of the esophagus late after Heller-Pinotti surgery, and another was suggested to undergo an esophagectomy, but the patient refused the procedure. Those patients who had dysphagia daily or at every meal were considered to have persistent dysphagia. No postoperative deaths were reported.

The most frequent symptoms in the preoperative period were persistent dysphagia (97.7%) and weight loss (68.1%), while in the postoperative period, they were 15.1% and 6.8%, respectively. It is noteworthy that 11 (33.3%) patients presented no symptoms in postoperative follow-up. The chi-square test was used to assess, in patients with more than one year of follow-up, the symptoms that had a reduction in the postoperative period at a level of statistical significance (p<0.05). The following symptoms had a significant reduction: persistent dysphagia, weight loss, regurgitation, and vomiting (p<0.05). The association of these categorical variables in the pre and postoperative period was assessed using Cramer’s V test, which has a range of 0–1, and the closer to 1, the greater the association between these variables (Table 3).

**Table 3:** Pre and postoperative symptoms.

Symptom	Preoperative n (%)	Postoperative n (%)	p<0.05	Cramer’s V test
Dysphagia	43 (97.7)	5 (15.1)	0.00	0.14
Regurgitation	24 (54.4)	2 (6.1)	0.00	0.08
Heartburn	7 (15.9)	5 (15.1)	0.92	-
Vomit	17 (38.6)	2 (6.1)	0.00	0.06
Odynophagia	11 (25.0)	4 (12.1)	0.15	-
Weight loss	30 (68.1)	3 (9.1)	0.00	0.10
None	0 (0)	11 (33.3)	-	-
Others	7 (15.9)	4 (12.1)	0.63	-

**Figure 1:**
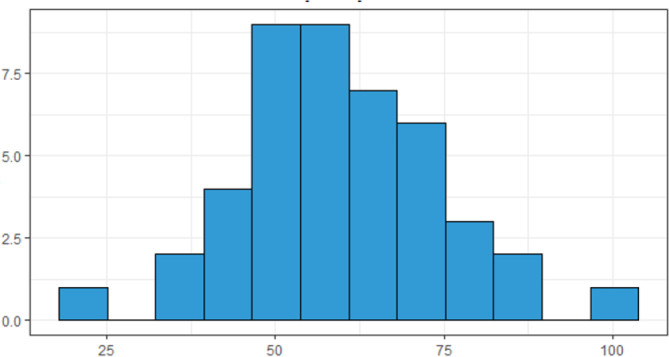
Histogram showing preoperative weight.

**Figure 2:**
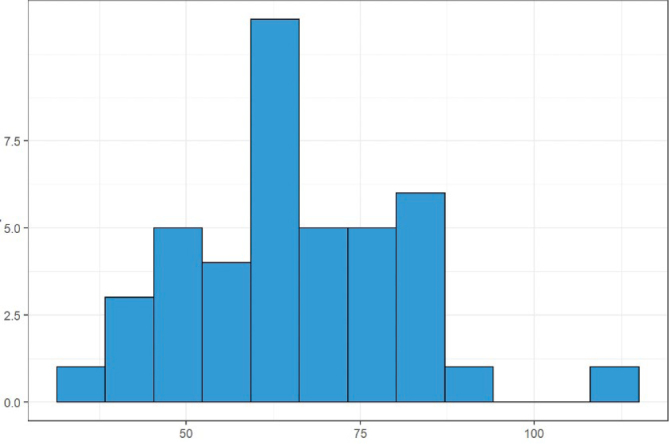
Histogram showing postoperative weight.

The mean preoperative weight was 59.4 kg (22.2–101 kg). The mean postoperative weight was 65.4 kg (38.3–115 kg), excluding two patients whose weight was not reported in the medical records (Figures 1 and 2).

**Figure 3:**
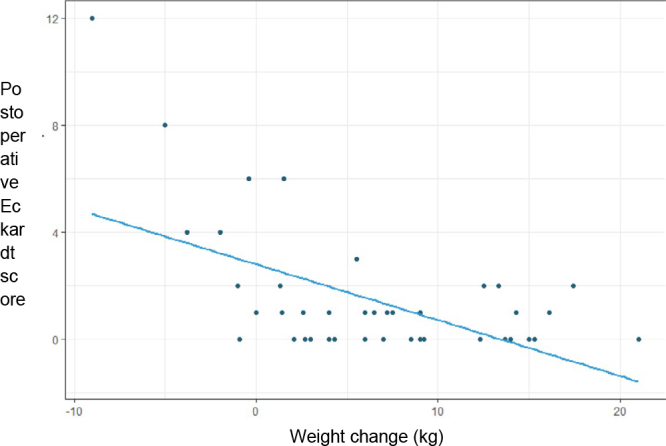
Eckardt score correlated with weight variation.

In the outpatient follow-up, 37 (84.1%) patients had an Eckardt score ≤3 indicating disease remission. The average Eckardt score was 6.5 preoperatively and 1.5 postoperatively. Eight patients were not considered for the calculation due to the lack of data in the medical records (Table 4). In the preoperative Eckardt scores, a median of 7 was observed with an interquartile range (IQR) of 5–8. In the postoperative, it was identified a median of 0.5 with an interval interquartile (IQI) of 0–1. Comparing the preoperative score with the postoperative, a median decrease of 6 points was found with an IQI of 3–7. The paired Wilcoxon test was performed to compare the pre and postoperative scores and, for this purpose, all patients from whom at least one of the two scores was not collected were disregarded. The test requires that the two samples have the same size, thus resulting in a sample size of 36 patients. Table 5 shows that based on the Wilcoxon test, there was a significant reduction in the Eckardt score postoperatively (p<0.05) (Table 5). Figure 3 demonstrates the inversely proportional correlation between weight gain or loss and the Eckardt scores in the postoperative period, evidencing the association of weight gain with the lowest Eckardt scores, and weight loss with the highest scores, confirmed by Pearson’s correlation coefficient of -0.557. This suggests that the greater the patient’s weight gain the lower his Eckardt score, and the greater the weight loss the higher the score.

**Table 4:** Mean Eckardt score.

Eckardt score	Average	Minimum	Maximum	Standard deviation
Preoperative	6.5	3	12	2,076.322
Postoperative	1.5	0	12	2,462.566

**Table 5:** Median Eckardt score (Paired Wilcoxon Test).

Score	Preoperative		Postoperative		Difference		p-value
	Median	IQI	Median	IQI	Median	IQI	
	7	5 to 8	0.5	0 to 1	-6	-7 to -3	p<10^-6^

IQI: interval interquartile range.

## DISCUSSION

Achalasia can affect both men and women, and according to some authors, it has an incidence of about 55% in men and 45% in women, showing a slight predominance in males, and mean age at diagnosis from 43.2 to 47.7 years^17,25,26^. In the present study, a slight predominance of males was also identified, with 52.3% and a mean age of 50.8 years. In our country, achalasia has Chagas disease as its main etiology, but it can also be due to idiopathic causes^20,33^. We identified in this sample 19 (43%) patients of idiopathic origin, a frequency that is higher than in other services in Brazil that presented from 11.6 to 30.7%^6,8,17,22,26^. All patients in this study had the chagasic etiology confirmed by two serological tests. The higher incidence of idiopathic achalasia can be justified due to the university hospital being considered one of the few reference centers for the treatment of megaesophagus in the region.

The study used the Rezende-Alves and Ferreira-Santos classifications, mainly considering the esophageal diameter to classify patients with non-advanced megaesophagus (grades I, II, and III)^14,34^. Half of the patients (22) corresponded to grade III as reported by Herbella et al.^17^ with 48.2%, and other authors reported a higher incidence of grade II (48) with 57.1%^17,23,26^.

Dysphagia is the most common symptom in patients with achalasia. The patients in the present study had an average dysphagia duration of 51 months (a little over four years), a time that is quite variable in other cases, which were 42.6, 74.4 and 102 months^9,17,23^. The average surgical time of the casuistic was 231 minutes — relatively high — and that can be explained due to the characteristic of a university hospital since surgical residents are in constant learning. In the reviewed literature, a study presented an average surgery time of 147 minutes^23^. The average length of hospital stay was 3.5 days, which is slightly longer than in the literature, probably because of the characteristics of patients admitted to the university hospital. Currently, the length of hospital stay is about 2.5 days reported in the literature^22^. The patients in this study had a mean postoperative follow-up time of 31.4 months, ranging 1–180 months and in other studies this time ranged 15.8–105^5,22,23,30^.

In the present study, 22.7% of patients had intraoperative complications — eight (18%) had esophageal perforations and two (4.5%) required conversion to laparotomy — while in the literature, no reports of conversions were found^5,9,22,23^. One conversion was due to severe hepatomegaly, leading to technical difficulties in performing the procedure, and another was due to hypercapnia, both at the beginning of the service’s series. All intraoperative esophageal perforations were diagnosed and treated simultaneously with mucosal suture, and none of these patients developed a fistula. Researchers reported a frequency ranging from 1.5–9.0% of mucosal perforation and these values were lower than those found in the present study^5,9,22,23^.

A current and controversial discussion is whether pneumatic endoscopic dilation increases the risk of mucosal perforation during surgery.

In this series, we had many patients who underwent preoperative dilation, making a total of 30 (68%) cases and many of these patients were submitted to more than one endoscopic dilation of the esophagus, which could justify a greater number of esophageal perforations. However, in the eight cases of esophageal perforation, five of them underwent preoperative endoscopic dilation, with a p-value of (p)=0.82, that is, no correlation between preoperative dilation and esophageal perforation. It is noteworthy that the study by Tsuboi et al. showed that preoperative dilation increased the incidence of postoperative reflux; despite this, it did not increase the number of intraoperative mucosal perforations^41^. Endoscopic dilation is reported as a therapeutic option with effective results in the treatment of achalasia, especially type II of the Chicago classification, as described by several authors^20,27,37,44,45^. However, the present study revealed 30 (68%) cases of patients who underwent preoperative dilations: eight had one dilation, ten had two, seven had three dilations, and four had more than three. All these cases required posteriorly Heller-Pinotti surgery, showing a high incidence of therapeutic failure of esophageal endoscopic dilation. In this study, no case of spleen injury, hemorrhages, or early complications was observed. Also, there was no case of death. The presence of gastroesophageal reflux and persistent postoperative dysphagia were considered as late complications (after one year of surgery), which is discussed below. We did not use the Chicago classification of achalasia because only three patients underwent high-resolution manometry due to the unavailability of the test at the hospital where the study was performed, but 32 patients underwent conventional manometry preoperatively.

All modalities of achalasia treatment can result in the onset of gastroesophageal reflux disease (GERD) but with different rates of involvement^1^. One of the criteria for evaluating the success of therapy for achalasia is the occurrence of GERD in a smaller number of patients. In this study, we considered postoperative gastroesophageal reflux as a late complication. Of the 33 patients evaluated after one year or more of follow-up, 9.0% evolved with reflux. GERD cases were diagnosed based on the clinical condition, which is considered a somewhat subjective situation for diagnosis. These patients required the use of proton pump inhibitors. The presence of GERD in post-treatment of achalasia is an issue that generates many discussions when we compare laparoscopic Heller myotomy with POEM, described for the first time in 2010 by Inoue et al.^18^. There are several studies with different results on this subject, such as the meta-analyses by Martins et al. and Inoue et al. which had similar results in the incidence of GERD when comparing these two techniques^24,40^. A recent meta-analysis by Campagna et al. showed 33.0% of GERD after POEM^3^. A recent case-control study showed that laparoscopic Heller myotomy and POEM are equally effective in clinical improvement of achalasia, although with high reflux rates in POEM^4^. Moura et al., in a recent prospective randomized study, showed a high rate of post-POEM reflux (64.6%) contrary to Heller-Pinotti surgery (11.1%)^10^. The casuistry presented here showed an incidence of 27.2% of long-term reflux, however with the caveat that not all these diagnoses were confirmed with digestive endoscopy and esophageal pHmetry. This may bias the result, as studies show approximately 10.0% of reflux post Heller-Pinotti surgery^10,36^.

The Heller surgery was performed for the first time in 1913 by the young German surgeon of the same name, performing anterior and posterior extramucosal esophagocardiomyotomy, and later, an anti-reflux valve was associated with Dor or Toupet^15,16^. A few years later, Pinotti et al. introduced a modification to the fundoplication by making three suture planes, one posteriorly and the other two at the edges of the myotomy, thus covering the mucosa^31,32^. This technique, known as the Heller-Pinotti surgery, is adopted by many surgical services in Brazil and was specifically evaluated in this study. In Scotland, in 1991, it was performed for the first time by videolaparoscopy, and more recently, it has been performed by robotic technique, maintaining favorable results^21,28,39^. The outcomes can be evaluated in several ways, and in the present study, we considered comparisons between pre and postoperative weight, the incidence of pre and postoperative symptoms and, more objectively, the postoperative reduction in the Eckardt score as criteria of successful treatment. These comparisons were made using data from the records of the last outpatient visit before surgery or data from the hospital admission for the procedure, compared with data from the last outpatient follow-up visit after surgery. In the preoperative period, the average weight was 59.4 kg (22.2–101 kg) and in the postoperative period was 65.4 kg (38.3–115 kg). From the two averages, it is possible to observe that the postoperative weight is higher than the preoperative one, as well as the maximum and minimum values. We can observe a greater number of patients with higher weights in the postoperative period when compared to the preoperative period.

These data may be due to improvement in dysphagia, as can be seen in the comparison of histograms in Figures 1 and 2. In 36 (81.8%) patients, weight gain was observed in the postoperative. A study by Csendes et al. reported weight gain in 87.5% of their patients, a result close to that found in the present series^9^. Persistent dysphagia was the most common preoperative symptom, affecting 97.7% of patients. In the postoperative period, the frequency of this symptom dropped to 15.1% in patients with more than one year of follow-up, in addition to the frequency of almost all other symptoms to a level of statistical significance. It is important to report that 11 (33.3%) patients presented with no symptoms after surgery. Studies in the literature have also shown a decrease in the incidence of all symptoms in the postoperative period and consequent improvement in quality of life, despite the fact that patients still had dysphagia and other symptoms sporadically after Heller-Pinotti surgery, as it is a functional disease that will probably never have a total remission^9,23,30,42^.

The Eckardt score was first described in 1992 to assess remission of achalasia after treatment with pneumatic dilation and later used to assess remission in other treatment modes. A score ≤3 means that the disease is under control^10,11,12,30^. The present study showed 37 (84.1%) patients with an Eckardt score ≤3. The mean score before surgery was 6.5 and after it was 1.5, resulting in a reduction of 5 points. The median Eckardt scores before and after surgery were 7 and 0.5, respectively, with an absolute difference of 6 points, showing a statistically significant reduction using the paired Wilcoxon test (p<0.000001). Paula et al.^30^ found an average reduction of 5 points in the Eckardt score, exactly the same as observed in the present study, besides several other researchers that showed a reduction, endorsing this data as a valid parameter of an effective result after laparoscopic Heller miotomy^5,10,30,43^. The current study showed, depending on the adopted criterion, a success rate in the treatment of achalasia from 81.8 to 86.4%, while other authors presented success rates of 80%, 81.7% and 100% as in the prospective study randomized trial by de Moura et al.^5,10,43^.

The present study has some limitations as it is a retrospective study with information taken from medical records that may lead to some bias in the results. Another limitation is that tests such as high-resolution manometry and esophageal pHmetry were not used to better assess patients.

## CONCLUSIONS

Laparoscopic esophagocardiomyotomy with fundoplication (Heller-Pinotti surgery) for the treatment of non-advanced achalasia (megaesophagus) is a safe and effective surgical technique. It has low rates of early and late complications with favorable results in improving the symptoms of the disease, and consequently, may contribute to improving the quality of life of patients.
